# Evaluation of sensitivity and specificity of CanPatrol™ technology for detection of circulating tumor cells in patients with non-small cell lung cancer

**DOI:** 10.1186/s12890-020-01314-4

**Published:** 2020-10-20

**Authors:** Jingyao Li, Yi Liao, Yaling Ran, Guiyu Wang, Wei Wu, Yang Qiu, Jie Liu, Ningyu Wen, Tao Jing, Haidong Wang, Shixin Zhang

**Affiliations:** 1 Department of Thoracic Surgery, Southwest Hospital, Army Medical University (Third Military Medical University), Chongqing, China; 2 SurExam Bio-Tech, Guangzhou Technology Innovation Base, 80 Lan Yue Road, Science City, Guangzhou, China; 3 Department of Clinical Laboratory, Center of Laboratory Medical, Southwest Hospital, Army Medical University (Third Military Medical University), Chongqing, China; 4 Department of Vasculocardiology, Southwest Hospital, Army Medical University (Third Military Medical University), Chongqing, China

**Keywords:** NSCLC, CTCs, CanPatrol™, Sensitivity, Specificity

## Abstract

**Background:**

The early diagnosis of non-small cell lung cancer is of great significance to the prognosis of patients. However, traditional histopathology and imaging screening have certain limitations. Therefore, new diagnostical methods are urgently needed for the current clinical diagnosis. In this study we evaluated the sensitivity and specificity of CanPatrol™ technology for the detection of circulating tumor cells in patients with non-small cell lung cancer (NSCLC).

**Methods:**

CTCs in the peripheral blood of 98 patients with NSCLC and 38 patients with benign pulmonary diseases were collected by the latest typing of CanPatrol™ detection technology. A 3-year follow-up was performed to observe their recurrence and metastasis. Kruskal-Wallis test was used to compare multiple groups of data, Mann-Whitney U test was used to compare data between the two groups, and ROC curve analysis was used to obtain the critical value. The COX risk regression and Kaplan-Meier survival analysis were performed in the 63 NSCLC patients who were effectively followed up.

**Results:**

The epithelial, epithelial-mesenchymal, and total CTCs were significantly higher in NSCLC patients than that in patients with benign lung disease (*P* <  0.001). The mesenchymal CTCs of NSCLC patients was slightly higher than that of benign lung diseases (*P* = 0.013). The AUC of the ROC curve of the total CTCs was 0.837 (95% CI: 0.76-0.914), and the cut-off value corresponding to the most approximate index was 0.5 CTCs/5 ml, at which point the sensitivity was 81.6% and the specificity was 86.8%. COX regression analysis revealed that the clinical stage was correlated with patient survival (*P* = 0.006), while gender, age, and smoking were not (*P* > 0.05). After excluding the confounders of staging, surgery, and chemotherapy, Kaplan-Meier survival analysis showed that patients in stage IIIA with CTCs ≥0.5 had significantly lower DFS than those with CTCs < 0.5 (*P* = 0.022).

**Conclusions:**

CTC positive can well predict the recurrence of NSCLC patients. CanPatrol™ technology has good sensitivity and specificity in detecting CTCs in peripheral blood of NSCLC patients and has a certain value for clinical prognosis evaluation.

**Supplementary information:**

**Supplementary information** accompanies this paper at 10.1186/s12890-020-01314-4.

## Background

The incidence and mortality of lung cancer rank first in all malignancies [1]. According to histological classification, lung cancer can be divided into non-small cell lung cancer (NSCLC) and small cell lung cancer (SCLC). NSCLC accounts for about 85% of lung cancer and the main subtypes are lung adenocarcinoma and lung squamous cell carcinoma [2, 3]. Although screening, early diagnosis and treatment can improve the survival rate of lung cancer patients, the low sensitivity of the currently approved low-dose CT scan screening leads to a false positive rate of over 90% [4]. There are currently no additional biomarkers to improve the sensitivity of low-dose CT screening, especially for patients with uncertain lung nodules. Besides, as main methods to diagnose and evaluate treatment efficacy of NSCLC, histopathology and imaging also have limitations. For example, there are certain restrictions in the actual operation of obtaining a tissue specimen for pathological examination with risking of bleeding, pneumothorax, and planting. Also, tissue biopsy is difficult to fully reflect the heterogeneity of the tumor, and cannot accurately predict the occurrence of drug resistance [5]. As for imaging examination, it is difficult to find small metastatic lesions, which is lagging in monitoring the efficacy of chemotherapy and the resistance of targeted drugs [6]. Therefore, new methods are urgently needed to remedy the current shortcomings to improve the screening, diagnoses and prognostic evaluation in lung cancer, and to achieve early prediction of treatment efficacy and dynamic monitoring of the condition.

Circulating tumor cells (CTCs) are tumor cells that enter the peripheral blood circulation spontaneously or by medical treatment caused. CTCs originate from the primary or metastatic tumor and can reflect the genetic information of the tumor in real time [7]. Studies have shown that the detection of CTCs contributes to the early diagnosis of NSCLC, as well as monitoring postoperative tumor recurrence and metastasis, and selecting individualized treatment strategies [8–10]. During the process of tumor cells detaching from the primary lesion into the blood circulation, some cells undergo epithelial-mesenchymal transition (EMT). Therefore, CTCs can be divided into epithelial CTCs, mesenchymal CTCs, and epithelial-mesenchymal CTCs [11]. During the EMT process, the expression of epithelial genes such as epithelial cell adhesion molecule (EpCAM) and cytokeratins (CK) is down-regulated, while the expression of mesenchymal genes such as vimentin and twist is up-regulated [12]. Studies have shown that a high proportion of mesenchymal CTCs predicted a worse prognosis for cancer patients, as well as a greater risk of metastasis, recurrence, and drug resistance [13, 14]. Therefore, further analysis of CTCs classification based on the number of CTCs is particularly important. By comparing both their changes, we can more comprehensively and accurately evaluate the tumor status, and achieve the accurate prognosis evaluation of NSCLC which will provide important information for the clinical treatment of NSCLC.

However, due to the scarcity of CTCs in the peripheral blood circulation and high individual heterogeneity, the sensitivity, specificity, and efficiency of CTCs detection technology are highly challenged. Most of the currently available methods on the market can only detect epithelial CTCs and epithelial-mesenchymal CTCs with epithelial markers. Even CellSearch®, a CTCs testing organization approved by the US FDA, also misses out on the more migratory and infiltrating mesenchymal CTCs [8]. In a previous study, the optimized CanPatrol CTC enrichment technique was used to classify CTCs by using EMT markers in different types of cancers [15]. Therefore, here, we provide a more comprehensive and systematic data to explore the sensitivity and specificity of the latest CanPatrol™ technology for detection of CTCs in peripheral blood of NSCLC patients.

## Methods

### Study subjects

A total of 136 patients who were admitted to the department of thoracic surgery of the first affiliated hospital of the Army Medical University from August 2015 to December 2015 were selected as the study subjects. The subject patients were diagnosed with NSCLC or pulmonary benign diseases through clinical manifestations, medical history, and pathology. All the enrolled patients had no history of other malignancies and did not receive related anti-tumor treatments before participation in our study. Before surgical treatment, the peripheral blood of subjects was sampled within 2 weeks before and after the imaging examination.

### Blood sampling and enrichment

Five milliliter peripheral blood was collected using a blood collection needle No. 8 (WEGO, Shangdong, China) and an EDTA-containing anticoagulation blood collection tube (WEGO, Shangdong, China). The following pretreatments were performed within 4 h after blood sample collection. Fifteen milliliter of erythrocyte lysis was firstly added into the sample and mixed well. Then, placed at room temperature for 30 min to allow the erythrocytes were fully lysed. After centrifugation for 5 min, the supernatant was discarded, 4 ml of PBS and 1 ml of RI fixative were added to fix the remain cells. The fixed cells were transferred to a filter tube containing an 8 μM pore size filter membrane (SurExam, Guangzhou, China), and filtered up using a vacuum pump (Auto Science, Tianjin, China). The filtered cell samples were further fixed at room temperature for 1 h by 4% formaldehyde.

### Multiple mRNAs in situ analysis

The fixed cell samples were treated with 0.1 mg/mL proteinase K to increase the cell membrane permeability. Next, specific capture probes (epithelial biomarker probe: EpCAM and CK8/18/19; mesenchymal biomarker probe: vimentin and twist; leukocyte marker: CD45) were added for hybridization. The sequences of these probes were listed in Supplementary Table 1. After incubating, the unbound probes were washed away with 0.1 × SSC eluent (Sigma, St. Louis, USA). Then incubated with the pre-amplification and the amplification solution to amplify the probe signal, and following incubated with three fluorescence-labeled probes at 40 °C. Namely, Alexa Fluor 594 (for epithelial biomarker probes EpCAM and ck8/18/19), Alexa Fluor 488 (for mesenchymal biomarker probes vimentin and twist) and Alexa Fluor 750 (for leukocyte marker CD45), and the sequences were listed in Supplementary Table 2. Finally, after staining nuclear with DAPI, the samples were observed using an automated fluorescence scanning microscope under 100x oil objective (Olympus BX53, Tokyo, Japan).

### Positive criterion

**Fig. 1:**
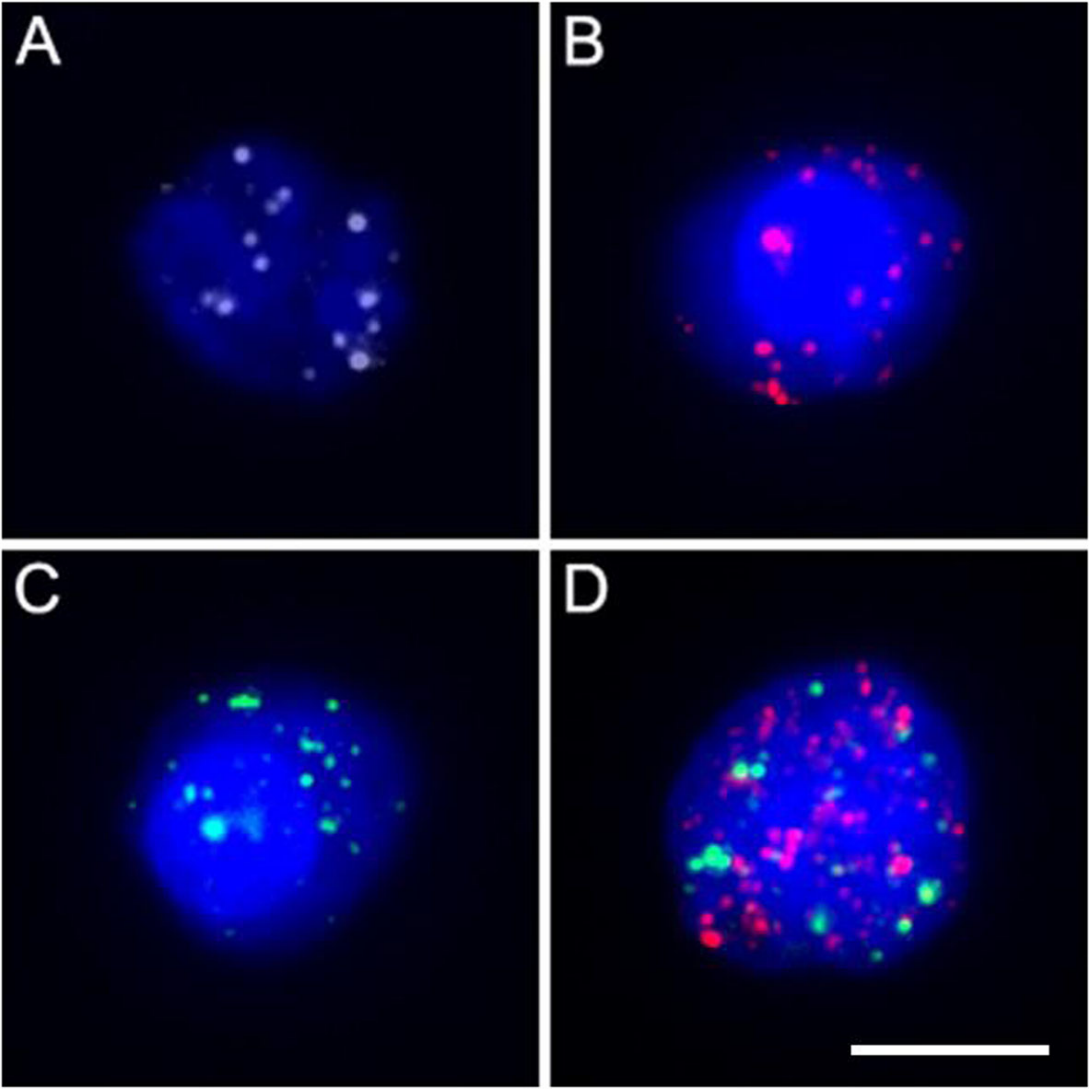
Fluorescence of CTCs. **a**. leukocyte. **b**. Type I CTCs (epithelial marker labeled, red fluorescence); **c** Type III CTCs (mesenchymal marker labeled, green fluorescence); **d**. Type II CTCs (epithelial and mesenchymal marker labeled, red and green fluorescence). Scale bar, 10 μm

The cell which has the number of fluorescence signal spot greater than or equal to 7 to be considered a valid count according to reagent instructions (SurExam, Guangzhou, China). The red fluorescence spot represents the epithelial marker expression and the green fluorescence spot represents the mesenchymal marker expression. Both red and green fluorescence was observed to represent the epithelial-mesenchymal type of CTCs (Table 1, Fig. 1).

**Table 1:** CTCs classification criteria

Type	Red spot	Green spot	Gray spot	DAPI
CTCs				
I	+	–	–	+
II	+	+	–	+
III	–	+	–	+

Type I: epithelial CTCs, red fluorescence

Type II: epithelial-mesenchymal CTCs, red and green fluorescence

Type III: mesenchymal CTCs, green fluorescence

### Follow-up

A total of 98 NSCLC patients who underwent radical surgery were followed up by telephone or clinic. The follow-up contents were chest CT, abdominal color Doppler ultrasound, skull MRI, whole-body bone scan, and PET-CT examination if necessary. The criteria for defining postoperative recurrence and metastasis in patients with lung cancer are imaging examinations suggesting that space-occupying lesions occur both inside and/or outside the lung. The follow-up period was 3 years and ended on December 31, 2018.

### Statistical analysis

Data analysis and charting were performed using SPSS 25.0 (IBM, USA). Because of the CTCs levels were significantly skewed, the Kruskal-Wallis test was used for comparison between multigroup while the Mann-Whitney U test was used for comparison between the two groups. The inspection level was α = 0.05. COX proportional hazard regression analysis was used to analyze the factors (staging, gender, age, and smoking) affecting patients’ survival, and the survival curve was plotted by the Kaplan-Meier method. The cut-off value was determined by the ROC curve.

## Results

### Patient characteristics

A total of 98 NSCLC patients were enrolled, including 65 males and 33 females, and the age distribution was between 18 and 82 years old (average age was 52 ± 9.3). There were 60 cases of lung adenocarcinoma, 33 cases of lung squamous cell carcinoma, and 5 cases of other NSCLCs. According to IASLC2009 (TNM staging standard for lung cancer, 2009, 7th edition), TNM staging was performed on the enrolled patients. Among them, 48 patients were stage I, 13 patients were stage II, 29 patients were stage III, and 8 patients were stage IV. There were 38 patients with benign lung diseases including 18 males and 20 females with the age distribution from 18 to 70 years (average age was 46 ± 11.7) (Table 2).

**Table 2:** Patients Characteristics and prevalence of circulating tumor cells

Characteristics	No.	CTCs (CTC Units/5 ml)											
		Epithelial CTCs			Mixed CTCs			Mesenchymal CTCs			Total CTCs		
		M	P25-P75	*P*	M	P25-P75	*P*	M	P25-P75	*P*	M	P25-P75	*P*
Benign lung diseases	38	0	0-0	< 0.01	0	0-0	< 0.01	0	0-0	0.013	0	0-0	< 0.01
NSCLC	98	1	0-2		1	0-3		0	0-1		3	1-6	
Pathological type				0.845			0.528			0.904			0.579
AC	60	1	0-2.75		1.5	0-3		0	0-1		3	1-6	
SC	33	1	0-2		1	0-2		2	0-0.5		2	1-5	
Others	5	1	0.5-3		2	0-3		1	0.5-1		2	1-7	
TNM stage				0.850			0.954			0.505			0.926
I	48	1	0-2		1	0-3		0	0-1		3	1-6	
II	13	1	0.5-3		1	0-4		0	0-1		3	1-11	
III	29	1	0-2.5		1	0-3		0	0-0.5		3	1-6	
IV	8	1.5	0-4.5		1	0.25-5		0.5	0-1.75		3	0.25-12.75	
Age													
≤ 60y	74	1	0-2	0.446	1	0-3	0.470	0	0-1	0.353	3	1-6	1
> 60y	24	1	0-2		0	0-3.75		0	0-0		2.5	0-6	

*Abbreviations*: *NSCLC* non-small cell lung cancer, *AC* Adenocarcinoma, *SC* Squamous carcinoma, *CTCs* circulating tumor cells, *M* median, *P25-P75* inter-quartile range

### Comparison of the number of CTCs between groups

The number of all subtypes of CTCs and the total number of CTCs in NSCLC were higher than those in the benign lung disease group (Mann-Whitney U test: The U value of epithelial CTCs group was 822.5, *P* <  0.01; the U value of epithelial-mesenchymal CTCs group was 859, *P* <  0.01; the U value of mesenchymal CTCs group was 1487, *P* = 0.013; and the U value of total CTCs was 605.5, *P* <  0.01). There was no statistically significant difference in the number of CTCs between lung adenocarcinoma, lung squamous cell carcinoma, and other NSCLC. According to the Kruskal-Wallis test, there was no statistically significant difference in the number of CTCs between TNM stages. Also, there was no significant difference in the number of CTCs between NSCLC patients at different ages (≦ 60 years or > 60 years) (Table 2). The detection rates of CTCs in stage I, II, III, and IV lung adenocarcinoma were 81, 80, 89, and 67%, respectively, while lung squamous cell carcinoma was 71, 100, 80, and 100%, respectively (Supplementary Table 3).

### ROC curve analysis to determine the cut-off value and assess the diagnostic performance

**Fig. 2:**
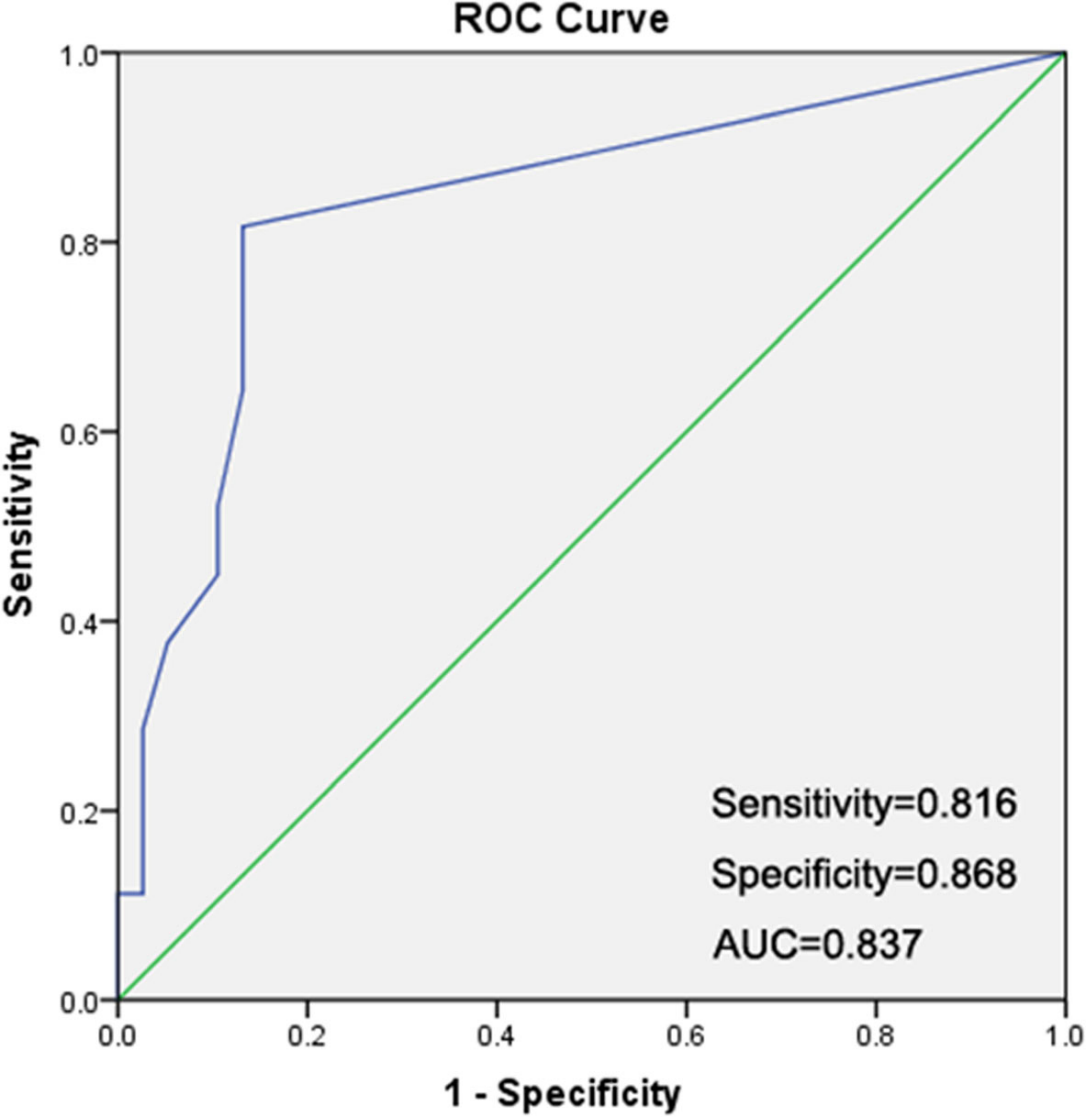
The ROC curve of CanPatrol™ technology-based CTCs of NSCLC. There were 38 benign patients, including 33 CTC negative and 5 CTC positive patients; and 98 NSCLC patients, including 18 CTC negative and 80 CTC positive patients

Taking the pathological results as standard, the ROC curve of the total number of CTCs in the NSCLC group was plotted to compare with those in the benign lung disease group (Fig. 2). The area under the curve (AUC) was 0.837, 95% CI was 0.76-0.914. The critical value corresponding to the maximum value of the Youden index was 0.5 CTC/5 mL. That was when the number of CTCs ≥0.5 was considered positive, the sensitivity was 81.6% and the specificity was 86.8%. Among them, the diagnostic sensitivity of stage I, II, III, and IV NSCLC was 79.2, 84.6, 86.2 and 75.0%, and the false-negative rate was 20.8, 15.4, 13.8, and 25.0%, respectively (Supplementary Table S4).

### COX proportional hazard regression analysis

A total of 63 of the 98 NSCLC patients were effectively followed up for 3 years. COX proportional hazard regression analysis revealed that the tumor stage was a risk factor for recurrence and metastasis in NSCLC patients (*P* = 0.006), while gender, age, and smoking were not risking factors for recurrence and metastasis (*P* > 0.05) (Table 3). The Exp(B) of tumor staging was 1.813, and the 95.0% CI was 1.186-2.772, indicating that for each upgrade of tumor stage, the risk of recurrence and metastasis was increased by 1.813times.

**Table 3:** COX proportional hazard regression analysis of follow-up information for 63 NSCLC patients

			95.0% CI for Exp (B)	
	*P*	Exp(B)	Lower	Upper
Stage	0.006	1.813	1.186	2.772
Smoking	0.843	0.895	0.299	2.680
Gender	0.745	0.820	0.248	2.709
Age	0.517	0.985	0.941	1.031

### The progress prediction ability of CTCs

The 63 followed-up patients were grouped according to the TNM stage, chemotherapy, pathological type, smoking, gender, and age. For each prognostic factor, the progress of the CTC ≥ 0.5 group has no difference from that of all patients (*P* > 0.05): TNM stage (*P* = 0.952), chemotherapy (*P* = 0.877), pathological type (*P* = 0.649), smoking (*P* = 0.968), gender (*P* = 0.61), age (*P* = 0.877), as shown in Supplementary Table S5.

### Kaplan-Meier survival analysis

**Fig. 3:**
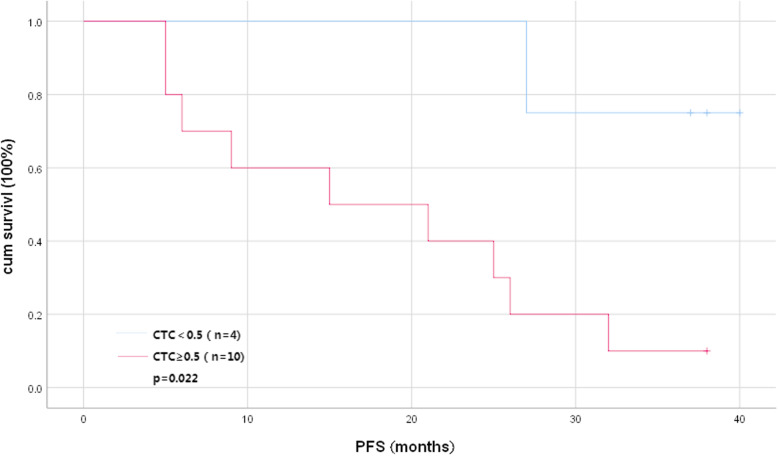
Survival curve of the stage IIIA NSCLC patients. The Kaplan-Meier curve shows the DFS of 14 patients with IIIA undergoing radical surgery and subsequent four rounds of adjuvant chemotherapy, stratified according to the total number of CTCs (CTCs ≥0.5, 10 cases and CTCs < 0.5, 4 cases)

Due to the close relationship between PFS and TNM staging as well as whether chemotherapy is performed, finally 14 stage IIIA patients of the followed-up 63 NSCLC patients met the same TNM staging and the same treatment conditions. The 14 patients who underwent radical surgery and subsequent four rounds of adjuvant chemotherapy were divided into two groups according to the total number of CTCs (CTCs ≥0.5, 10 cases and CTCs < 0.5, 4 cases). Kaplan-Meier survival analysis results showed that the DFS (progression-free survival) of patients with the total number of CTCs ≥0.5 was significantly lower than that of patients with the total number of CTCs < 0.5 (*P* = 0.022) (Fig. 3).

## Discussion

CTCs refers to tumor cells released into the peripheral blood by primary tumors and/or metastatic lesions. Because CTCs are important to the formation of metastasis, and they are highly implicated in tumor-related deaths. Therefore, the detection of CTCs in peripheral blood is important for early diagnosis and for efficacy and prognosis evaluation [8–10, 16]. However, due to the very limited number of CTCs in peripheral blood circulation, the heterogeneity of CTCs subtypes, and the easy aggregation into micro-plugs etc., the sensitivity, specificity, and efficiency of CTCs detection technology are extremely challenged [17].

The key steps for CTCs detection are enrichment and identification. Currently, CTCs are sorted from other cells in the blood mainly through physical characteristics (such as the size, density, chargeability and deformability of CTCs, etc.) and biological characteristics (such as the cell surface antigen) [18]. Sorting CTCs according to physics characteristics is simple in operation and relatively low in cost, but cannot avoid the interference of individual heterogeneity, while sorting CTCs according to biological characteristics ensures the accuracy, but is limited by the types of cell surface-expressed antigen. CTCs identification techniques include cell counting which is based on flow cytometry and nucleic acid detection which is based on a reverse transcriptase-polymerase chain reaction. Cell counting method can quantitatively detect the number of CTCs and analyze various parameters of the CTCs (such as the size, morphology, intracellular and extracellular biomarkers, as well as the genomic mutations), but the detection sensitivity is low and requires a large volume of blood sample; The advantages of the nucleic acid detection method are time-saving, highly specific and requiring fewer blood samples, but this process inevitably destroys cell morphology and function, making further analysis impossible. In addition, due to the easy degradation of mRNA and the influence of non-specific amplification, the false positive rate increases [18–21]. The CellSearch system is currently widely recognized and used in the detection of lung cancer CTCs, which consists mainly of automated immunomagnetic separation systems and immunofluorescence analysis systems. The CTCs are isolated and enriched based on the EpCAM expression, but mesenchymal CTCs that had undergone epithelial-mesenchymal transformation could not be detected [8]. Therefore, currently, there is no ideal method for detecting CTCs in the peripheral blood of NSCLC patients.

The CanPatrol™ technology used in this study combined nanomembrane filtration technology and multiple RNA in situ analysis techniques to sort and identify CTCs. Canpatrol™ CTC detection technology (Canpatrol™, Surexam) effectively overcomes the limitations of only isolating a specific epithelial phenotype of CTC and missing the detection of leukocyte-CTC cell clusters. CTCs are retained by nano-membrane filtration and analyzed the specific genes by highly sensitive multiple RNA in situ analysis (MRIA). Accurate classification of human peripheral blood CTCs was achieved. It contains five types including epithelial CTCs, mesenchymal CTCs, epithelial-mesenchymal CTCs, cluster CTCs, and leukocyte-CTCs cluster. We used nanomembrane with a self-optimized pore size of 8um to filter peripheral blood so that the tumor cells in the peripheral blood were highly enriched. Previous studies have shown that the enrichment rate was as high as 89%, and the leukocyte removal rate was as high as 99.98% [22]. The advantage of this method is that it can completely sort all types of CTCs (epithelial, epithelial-mesenchymal and mesenchymal CTCs) without relying on specific biomarkers, and could be applied to enrich most of the solid tumors’ CTCs [15]. In addition, Canpatrol™ adopts a novel multiple mRNAs in situ analysis method to hybridized the specific probes to the target gene and further enhance the sensitivity and specificity of the detection through the fluorescence signal cascade amplification system. In this study, we compared CTCs in peripheral blood of patients with NSCLC and benign lung diseases. Statistical analysis showed that there were differences in the number of three subtypes of CTCs and total CTCs between the two groups. ROC curve analysis showed that the sensitivity and the specificity of CanPatrol™ technology for the detection of peripheral blood CTCs in NSCLC was 81.6 and 86.8%, respectively. It can be concluded that this method has better diagnostic accuracy for NSCLC and has obvious diagnostic advantages compared with other methods. Additionally, as a non-specific physical enrichment technology, Canpatrol™ reduces the damage of tumor cells in peripheral blood preserving the original cellular information, such as morphology, cell function, molecular biology information, etc. Therefore, Canpatrol™ technology is beneficial for subsequent immunofluorescence, fluorescence in situ hybridization (FISH), gene expression, gene mutation detection, and microdissection based single-cell sequencing analysis of CTCs. Moreover, this technology can also be used for cell culture and animal models to develop new drugs and conduct the drug susceptibility testing, which would comprehensively and dynamically reveal tumor molecular information and guide the individualized treatment for cancer patients.

In this study, there was no statistically significant difference in the number of CTCs between lung adenocarcinoma, lung squamous cell carcinoma, and other NSCLCs which is consistent with previous studies [23, 24]. CTC is mainly to predict the risk of recurrence and metastasis and to evaluate the efficacy. There is not much correlation with the pathological type. This conclusion is in accordance with others studies [25, 26]. As for whether there is a difference, is it because the number of cases is not enough to obtain an accurate conclusion, more studies are needed to confirm the correlation between staging and CTC. There was no statistical difference in the number of subtype CTCs and total CTCs between different ages (≦ 60 years or > 60 years), indicating that age is not a factor influencing CTCs, and our result is consistent with previous studies [23, 24, 27]. Through COX proportional hazard regression analysis of the follow-up data, we found that pathological stage is a risk factor for recurrence and metastasis which indicating that it is more scientific to plot the survival curve after risk screening and stratification. The results of 63 follow-up patients showed that the number of metastases in CTC-positive patients accounted for most of the total number of metastases. Therefore, we believe that CTC can be used as an auxiliary method for clinical prognosis of lung cancer. According to the ROC curve analysis and the cut-off value, the number of CTCs ≥0.5 was judged as positive. After a survival analysis of 14 patients with stage IIIA, we concluded that patients with NSCLC with a total number of CTCs ≥0.5 have significantly lower DFS than patients with number < 1, which is consistent with previous reports [23, 28]. Our data suggest that the number of total CTCs ≥0.5 in peripheral blood (5 ml) of NSCLC patients could predict the prognosis. However, it is necessary to expand the number of cases and extend the follow-up time to verify this conclusion.

## Conclusions

In summary, CanPatrol™ has high sensitivity and specificity in detecting peripheral blood CTCs in NSCLC patients, which is of a certain value in clinical diagnosis and prognosis.

## Supplementary information

**Additional file 1: Supplementary Table 1.** Capture probe sequences. **Supplementary Table 2.** Sequences for the bDNA signal amplification probes. **Supplementary Table 3.** CTC Detection rate in TNM stages among NSCLC patients with different pathological types. **Supplementary Table 4.** Diagnostic sensitivity and false negative of NSCLC based on cut-off value of CTCs. **Supplementary Table S5.** Prognosis of NSCLC based on cut-off value of CTCs. (DOCX 55 kb)

## Data Availability

The dataset supporting the conclusions of this article is included within the article’s additional file.

## Abbreviations

CK: Cytokeratins
CTCs: Circulating tumor cells
EMT: Epithelial-mesenchymal transition
EpCAM: Epithelial cell adhesion molecule
FISH: Fluorescence in situ hybridization
NSCLC: Non-small cell lung cancer
SCLC: Small cell lung cancer

## References

[1] Bray F, Ferlay J, Soerjomataram I, Siegel RL, Torre LA, Jemal A (2018). Global cancer statistics 2018: GLOBOCAN estimates of incidence and mortality worldwide for 36 cancers in 185 countries. CA Cancer J Clin.

[2] Herbst RS, Heymach J, Lippman SM (2008). Lung Cancer. N Engl J Med.

[3] Gridelli C, Rossi A, Carbone DP, Guarize J, Karachaliou N, Mok T, Petrella F, Spaggiari L, Rosell R (2015). Non-small-cell lung cancer. Nat Rev Dis Primers.

[4] Aberle DR, Adams A, Berg CD, Black WC, Clapp JD, Fagerstrom RM (2011). Reduced lung-cancer mortality with low-dose com-puted tomographic screening. N Engl J Med.

[5] Esposito A, Criscitiello C, Locatelli M, Milano M, Curigliano G (2016). Liquid biopsies for solid tumors: understanding tumor heterogeneity and real time monitoring of early resistance to targeted therapies. Pharmacol Ther.

[6] Ettinger DS, Akerley W, Borghaei H, Chang AC, Cheney RT, Chirieac LR (2012). Non-small cell lung cancer. J Natl Compr Cancer Netw.

[7] O'Flaherty JD, Gray S, Richard D, Fennell D, O'Leary JJ, Blackhall FH, O'Byrne KJ (2012). Circulating tumour cells, their role in metastasis and their clinical utility in lung cancer. Lung Cancer.

[8] Tartarone A, Rossi E, Lerose R, Mambella G, Calderone G, Zamarchi R, Aieta M (2017). Possible applications of circulating tumor cells in patients with non small cell lung cancer. Lung Cancer.

[9] Krebs MG, Sloane R, Priest L (2011). Evaluation and prognostic significance of circulating tumor cells in patients with non-small-cell lung cancer. J Clin Oncol.

[10] Hou JM, Krebs M, Ward T, Sloane R, Priest L, Hughes A, Clack G, Ranson M, Blackhall F, Dive C (2011). Circulating tumor cells as a window on metastasis biology in lung cancer. Am J Pathol.

[11] Ksiazkiewicz M, Markiewicz A, Zaczek AJ (2012). Epithelial-mesenchymal transition: a hallmark in metastasis formation linking circulating tumor cells and cancer stem cells. Pathobiology.

[12] Raghu K (2009). EMT: when epithelial cells decide to become mesenchymal-like cells. J Clin Invest.

[13] Liu H, Zhang X, Li J, Sun B, Qian H, Yin Z (2015). The biological and clinical importance of epithelial-mesenchymal transition in circulating tumor cells. J Cancer Res Clin Oncol.

[14] Lowes LE, Allan AL (2018). Circulating tumor cells and implications of the epithelial-to-Mesenchymal transition. Adv Clin Chem.

[15] Wu S, Liu S, Liu Z, Huang J, Pu X, Li J, Yang D, Deng H, Yang N, Xu J (2015). Classification of circulating tumor cells by epithelial-mesenchymal transition markers. PLoS One.

[16] Cohen SJ, Punt C, Iannotti N (2008). Relationship of circulating tumor cells to tumor response, progression-free survival, and overall survival in patients with metastatic colorectal cancer. J Clin Oncol.

[17] Ferreira MM, Ramani VC, Jeffrey SS (2016). Circulating tumor cell technologies. Mol Oncol.

[18] Yu N, Zhou J, Cui F, Tang X (2015). Circulating tumor cells in lung cancer: detection methods and clinical applications. Lung.

[19] Adan A, Alizada G, Kiraz Y, Baran Y, Nalbant A (2017). Flow cytometry: basic principles and applications. Crit Rev Biotechnol.

[20] Alix-Panabieres C, Pantel K (2014). Technologies for detection of circulating tumor cells: facts and vision. Lab Chip.

[21] Chikaishi Y, Yoneda K, Ohnaga T, Tanaka F (2017). EpCAM-independent capture of circulating tumor cells with a 'universal CTC-chip. Oncol Rep.

[22] Wu S, Liu S, et al. Enrichment and enumeration of circulating tumor cells by efficient depletion of leukocyte fractions. Clin Chem Lab Med. 2014;52(2). 10.1515/cclm-2013-0558 24021598

[23] Murlidhar V, Reddy RM, Fouladdel S, Zhao L, Ishikawa MK, Grabauskiene S, Zhang Z, Lin J, Chang AC, Carrott P (2017). Poor prognosis indicated by venous circulating tumor cell clusters in early-stage lung cancers. Cancer Res.

[24] Liu DG, Xue L, Li J, Yang Q, Peng JZ (2018). Epithelial-mesenchymal transition and GALC expression of circulating tumor cells indicate metastasis and poor prognosis in non-small cell lung cancer. Cancer Biomark.

[25] Li S, Chen Q, Li H, Wu Y, Feng J, Yan Y. Mesenchymal circulating tumor cells (CTCs) and OCT4 mRNA expression in CTCs for prognosis prediction in patients with non-small-cell lung cancer. Clin Transl Oncol. 2017;19(9):1147–53. 10.1007/s12094-017-1652-z 28374320

[26] Li TT (2015). Evaluation of epithelial-mesenchymal transitioned circulating tumor cells in patients with resectable gastric cancer: relevance to therapy response. World J Gastroenterol.

[27] Tanaka F, Yoneda K, Kondo N, Hashimoto M, Takuwa T, Matsumoto S, Okumura Y, Rahman S, Tsubota N, Tsujimura T (2009). Circulating tumor cell as a diagnostic marker in primary lung cancer. Clin Cancer Res.

[28] Li Y, Cheng X, Chen Z, Liu Y, Liu Z, Xu S (2017). Circulating tumor cells in peripheral and pulmonary venous blood predict poor long-term survival in resected non-small cell lung cancer patients. Sci Rep.

