# Supplementary material for: Evaluation of sensitivity and specificity of CanPatrol™ technology for detection of circulating tumor cells in patients with non-small cell lung cancer
Source: BMC Pulm Med. 2020 Oct 20;20:274. doi: 10.1186/s12890-020-01314-4 (PMC7576719; doi:10.1186/s12890-020-01314-4)
Supplement: Supplementary file 1 — Additional file 1: Supplementary Table 1. Capture probe sequences. Supplementary Table 2. Sequences for the bDNA signal amplification probes. Supplementary Table 3. CTC Detection rate in TNM stages among NSCLC patients with different pathological types. Supplementary Table 4. Diagnostic sensitivity and false negative of NSCLC based on cut-off value of CTCs. Supplementary Table S5. Prognosis of NSCLC based on cut-off value of CTCs. (DOCX 55 kb) [file 12890_2020_1314_MOESM1_ESM.docx]

**Supplementary Table 1.** Capture probe sequences.


**Supplementary Table 2.** Sequences for the bDNA signal amplification probes.

(1) * Repeat the sequence once

(5) ** Repeat the sequence five times

**Supplementary Table 3.** CTC Detection rate in TNM stages among NSCLC patients with different pathological types.

Abbreviations: E – CTCs, Epithelial CTCs; M – CTCs, Mesenchymal CTCs; AC, Adenocarcinoma; SC, Squamous carcinoma.

**Supplementary Table 4.** Diagnostic sensitivity and false negative of NSCLC based on

cut-off value of CTCs.

Abbreviations: AC, Adenocarcinoma; SC, Squamous carcinoma.

**Supplementary Table S5.** Prognosis of NSCLC based on cut-off value of CTCs.
